# Childhood Interstitial Lung Disease Masquerading as Post COVID-19 Respiratory Distress

**DOI:** 10.7759/cureus.20061

**Published:** 2021-11-30

**Authors:** Sinan Yavuz, Ronda Alsamhouri, Nader Francis

**Affiliations:** 1 Paediatrics, Al Qassimi Women's and Children's Hospital, Sharjah, ARE; 2 Pediatrics, Al Qassimi Women`s and Children's Hospital, Sharjah, ARE; 3 Pediatric Pulmonology, Al Qassimi Women`s and Children`s Hospital, Sharjah, ARE

**Keywords:** infant respiratory distress, pediatric pulmonary disease, surfactant dysfunction, abca3 deficiency, childhood interstitial lung disease

## Abstract

Childhood interstitial lung diseases (chILD) are a set of illnesses affecting the bronchoalveolar spaces and the cellular compartment of the lungs. In the neonatal period, they are mainly classified under disorders of development, growth, surfactant dysfunction, and others of unknown causes distinctive in infancy. One of the most common causes is the deficiency of triphosphate binding cassette transporter A3 (ABCA3) protein. It activates impairment in the function of surfactants, resulting in respiratory distress in term infants, which is lethal in many cases and in some other cases leads to interstitial lung disease.

We herein present a case of a 14-month-old boy with a peculiar case of ABCA3 protein deficiency that was masked at birth with COVID-19 infection and then presented with shortness of breath and poor feeding at the age of three months. The child was treated with macrolides, steroids, and hydroxychloroquine, with which he survived beyond the age of one year.

## Introduction

Childhood interstitial lung disease (chILD) is a rare genetically inherited group of diseases that causes acute or chronic respiratory signs and findings. Etiologies are often unknown. In the neonatal period, they are mainly classified under disorders of development, growth, surfactant dysfunction, and others of unknown causes distinctive in infancy [1]. Pulmonary surfactant is a combination of proteins and lipids whose function is to keep the lung alveoli open and prevent them from collapsing. Type II pneumocytes produce them, and the lamellar bodies are responsible for their synthesis and metabolism. ABCA3 is a transporter found in the membrane in lamellar bodies and is involved in the biosynthesis of surfactants. Deficiencies of ABCA3 protein cause dysfunction in surfactants which results in respiratory distress syndrome in term babies as well as interstitial lung disease [2]. Homozygous mutations of ABCA3 protein cause complete loss in the surfactant function hence increased mortality within the first few months of life if lung transplantation was not achieved [3].

We report a case of a 14-month-old child who survived beyond the age of one year with a diagnosis of ABCA3 protein deficiency after being referred to us with a diagnosis of post-viral (Covid) bronchiolitis obliterans.

## Case presentation

**Figure 1:**
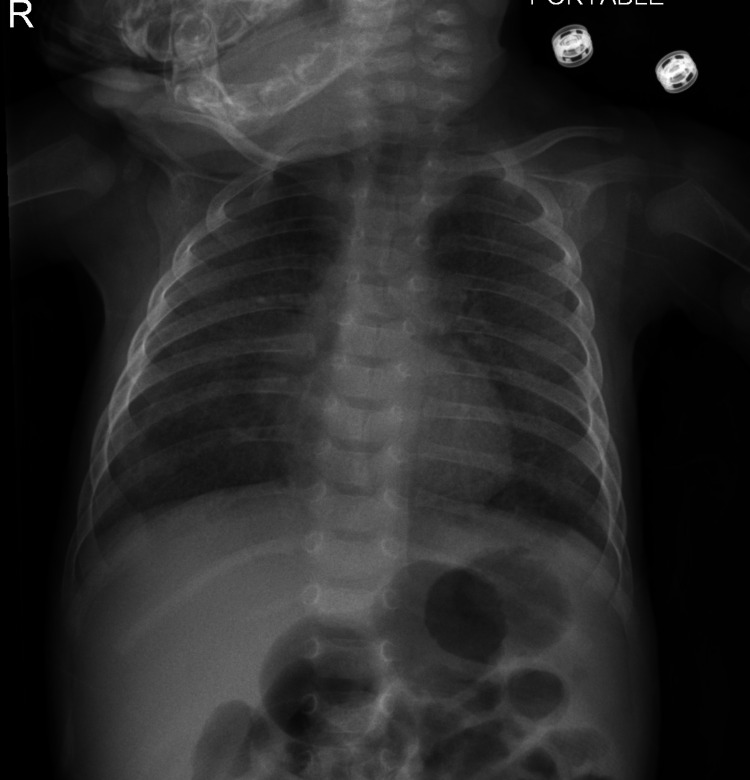
Chest X-ray revealed bilateral opacities at mid and lower lung zones.

A 14- month-old boy was referred to our hospital at the age of three months with the diagnosis of post-COVID-19 bronchiolitis obliterans for further management. The child was born at 39 weeks to a COVID-19 positive mother and consanguineous parents by normal vaginal delivery with a birth weight of 3.7 kg. He neither required any resuscitation at birth nor did he develop any respiratory distress. On day 2 of life, the baby tested positive for COVID-19 and was admitted for nine days. His investigations were found to be within normal ranges. He was kept on nasal cannula support for four days only. Three months after discharge, the child was readmitted due to difficulty breathing, cough, and poor feeding. Lab investigations were normal, with a negative respiratory viral panel. Initial chest X-ray showed bilateral opacities at mid and lower lung zones (Figure 1).

**Figure 2:**
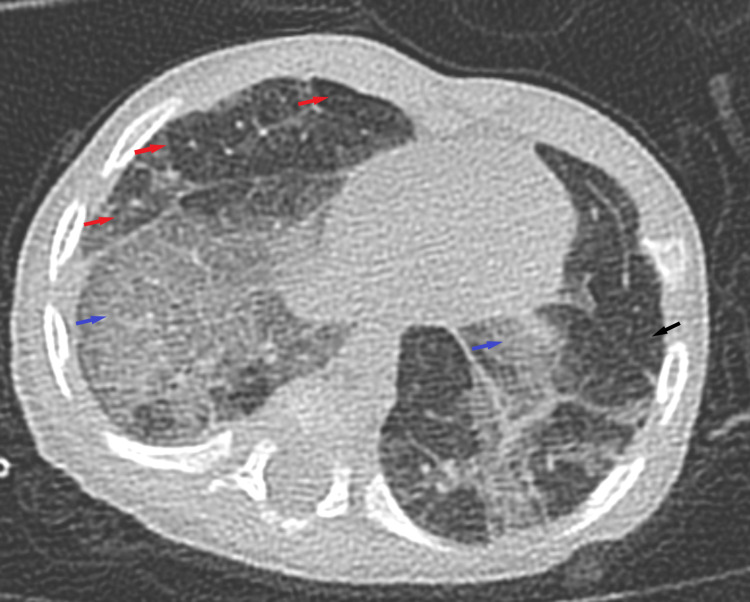
Chest CT scan with contrast. Chest CT with contrast showed mosaic attenuation pattern in both lower lungs (red arrows), bilateral ground-glass opacities with areas of reduced attenuation (blue arrows), areas of oligemia, and air-trapping (black arrow).

**Figure 3:**
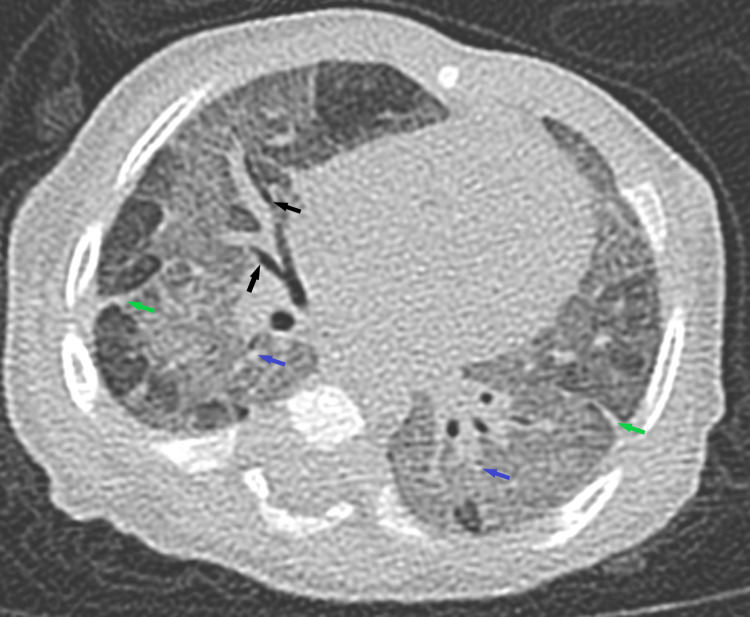
Chest CT scan with contrast. CT scan of the chest with contrast showed cylindrical bronchiectasis, mild bronchial wall thickening in areas of ground-glass attenuation, fibrotic changes with distorted lung architecture, and few mediastinal lymph nodes.

He was initially treated as post-COVID-19 bronchiolitis with possible superimposed infection, for which he received a course of antibiotics. Furthermore, as the child’s condition was not improving, further imaging studies were obtained. A CT scan of the chest with contrast showed mosaic attenuation pattern in both lower lungs, bilateral ground-glass opacities with areas of reduced attenuation, areas of oligemia and air-trapping, cylindrical bronchiectasis, mild bronchial wall thickening in areas of ground-glass attenuation, fibrotic changes with distorted lung architecture, and few mediastinal lymph nodes, all of these were features suggestive of bronchiolitis obliterans (Figures 2-3).

The baby was referred to our hospital with an impression of post-infectious bronchiolitis obliterans (PIBO) on oxygen support and fluticasone, azithromycin, and montelukast (FAM) regime.

On admission, further history was taken. Parents mentioned that the child was always having fast breathing with sweating and brief cyanotic episodes after feeding. On physical examination, a hypotonic and poorly nourished child with weight below fifth centile, chest examination showed mild tachypnea with subcostal and intercostal recession, bilateral fine crackles, other systems examinations were unremarkable. Initial investigations like complete blood count (CBC), electrolyte, liver, and kidney function tests as well as inflammatory markers were unremarkable. The child remained oxygen dependent with frequent desaturation below 75% and required admission to the intensive care unit (ICU) due to respiratory distress. At this time, in view of his clinical progression, review of the radiological findings, and lab tests, suspicion of interstitial lung disease was raised. Keeping in mind the differential diagnosis of the interstitial lung disease, we extended our scope of investigations. Flexible bronchoscopy was carried out with normal morphology. Bronchoalveolar lavage (BAL) showed many foamy macrophages with dispersed bronchial epithelial cells and degenerated epithelial cells mixed with inflammatory cells, mainly neutrophils with negative culture. The child was evaluated further with a cardiac echocardiogram, swallow study with the fiberoptic laryngoscope, and ultrasonography of the urinary system (US KUB), all of which were normal except for a right ectopic kidney on the US.

Further genetic evaluation for childhood interstitial lung diseases (chILD) panel was sent, which revealed a homozygous pathogenic variant mutation in the ABCA3 gene. The child was started on oral steroids, but he did not improve. He was admitted multiple times with aspiration pneumonia. He remained on 4L oxygen at home by nasal cannula.

**Figure 4:**
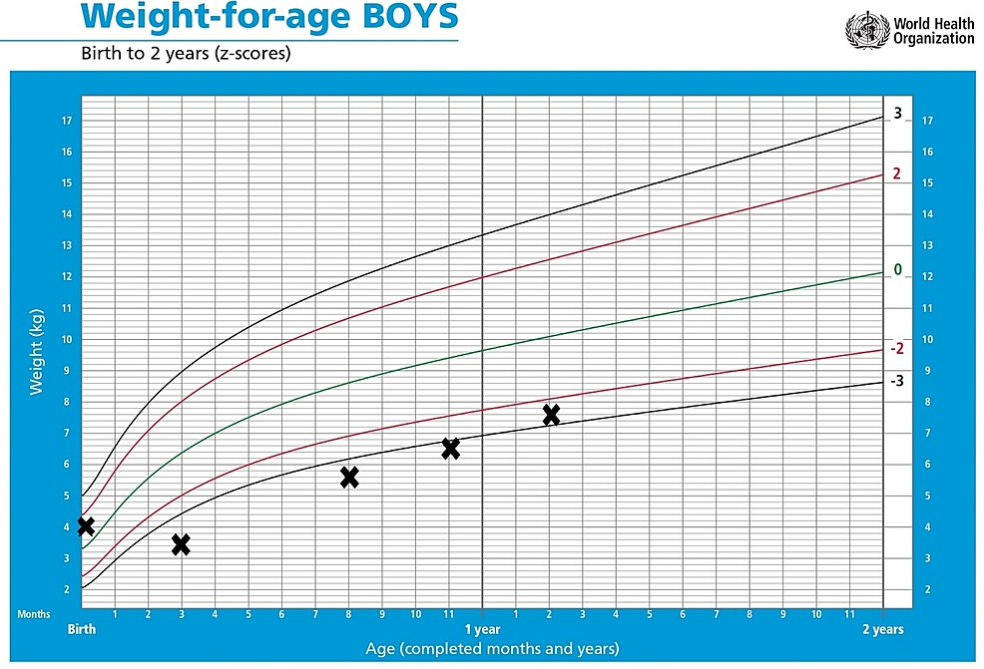
Changes in the weight of the patient over time, plotted on the standardized WHO weight-for-age growth chart for boys.

**Figure 5:**
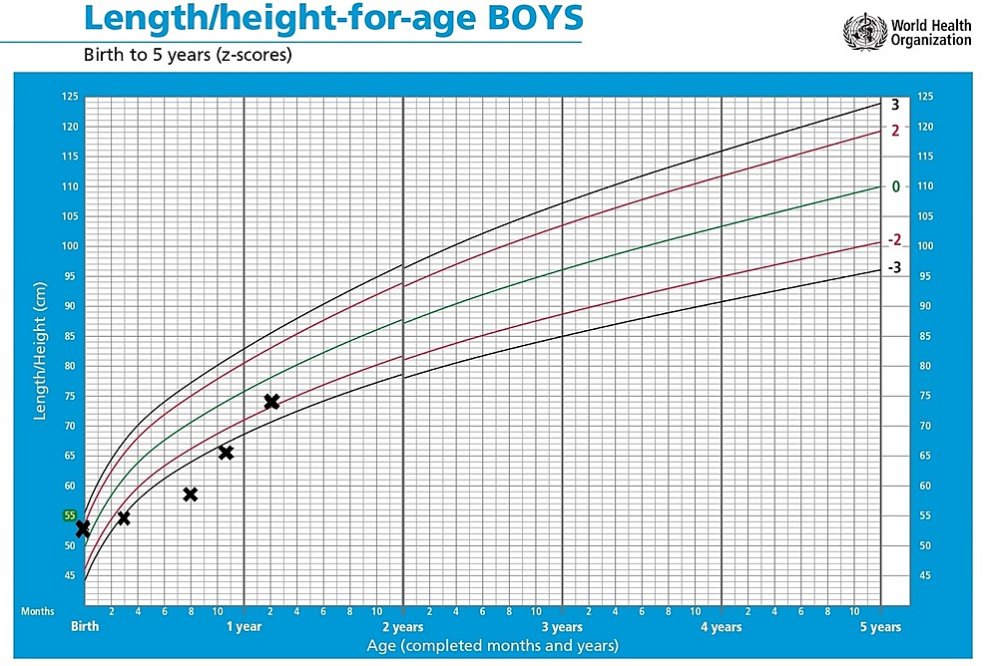
Changes in the weight of the patient over time, plotted on the standardized WHO length/height-for-age growth chart for boys.

At the age of seven months, as the child had a poor response to steroids, we decided to start a course of hydroxychloroquine 45 mg daily with azithromycin restarted 10 mg/kg/day for three days per week. Later, at the age of 13 months, the child was also started on methylprednisolone pulse therapy as 10 mg/kg/day for three days monthly. Currently, the child is 14 months old, gaining weight and height, on 0.5 L oxygen at home, and can last a couple of hours during the day without oxygen support (Figures 4-5).

**Figure 6:**
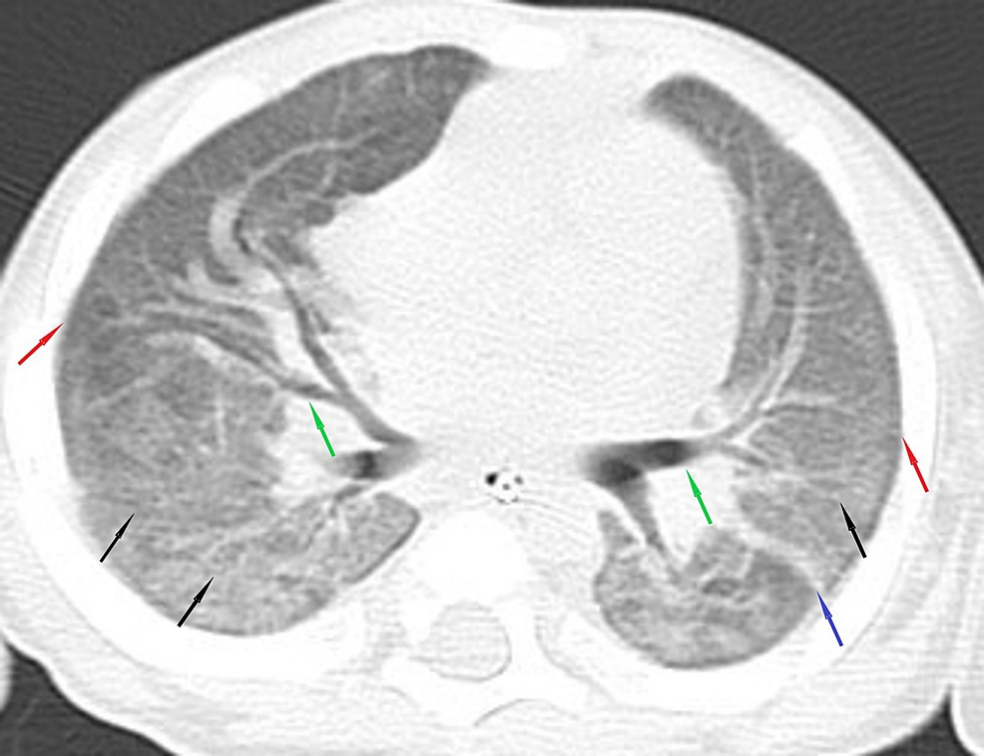
High-resolution chest CT scan. High-resolution chest CT scan revealed diffuse ground-glass opacities (black arrows), septations (blue arrow), central bronchiectasis (green arrows), subpleural small cysts (red arrows), and fibrotic changes.

The repeated high-resolution CT of the chest (HRCT) revealed diffuse ground-glass opacities, septations, central bronchiectasis, subpleural small cysts, and fibrotic changes, which did not show any progression from previous imaging (Figure 6).

We plan to continue on combination therapy with hydroxychloroquine, azithromycin, and methylprednisolone pulse therapy.

## Discussion

Childhood interstitial lung diseases are a set of disorders that affect the interstitium of the lung. Clinically, the child is presenting with tachypnea, cough, and oxygen dependency. Diagnosis is usually made with HRCT, bronchoscopy, genetic testing, and lung biopsy [4].

Surfactants are multiplex compounds that are made up of proteins and lipids. They are produced in type II pneumocytes and stored in lamellar bodies found within these cells. Once transported out of the cell into the alveolar lumen, they produce a mesh-like product named tubular myelin, which helps keep the alveoli from collapsing. In preterm infants, the deficiency of surfactants is due to the immaturity of type II alveolar cells, causing respiratory distress at birth. On the other hand, deficiencies of surfactants in term babies are usually due to mutations in their transcription, production, secretion, or transportation. The most common cause of surfactant dysfunction is mutations of the ATP-binding cassette subfamily A member 3 (ABCA3) genes. The ATP-binding cassette is a transporter group that encodes for genes that control the glycosylation, packaging, and secretion of the surfactant. The most common type is a homozygous mutation of c.4545C>G, which often is lethal within the first three months of life if lung transplantation was not done [3]. ABCA3 mutations have also been recognized as a cause of ChILD [2].

There are no clear guidelines for the treatment of interstitial lung disease caused by ABCA3 mutation yet. Corticosteroids were shown to upregulate the expression of ABCA3 in type II pneumocytes [5]. In addition, several studies have proven that a combination of corticosteroids, hydroxychloroquine (HCQ), and macrolides like azithromycin have been effective in interstitial lung disease with genetic etiologies including genetic mutations but was still suggested to be tried through empirical observation in children with ABCA3 mutations [5].

## Conclusions

In our case, the child’s diagnosis of interstitial lung disease was masked by the child’s infection with COVID-19 during the first week of life as there is an overlap between clinical pictures of ILD and bronchiolitis obliterans. Interestingly, despite the presence of homozygous mutation of ABCA3 protein, which is usually fatal in most documented cases, this child’s clinical course and oxygen requirement improved with the given treatment. The child survived past the suggested mortality age associated with this mutation. Given the mild to moderate improvement evident after the initiation of hydroxychloroquine and pulse steroid therapy in our patient, we believe that the prognosis of homozygous mutations of the ABCA3 gene may be more variable according to the mutation than previously suggested, especially with early diagnosis and proper treatment while bridging to lung transplantation.
